# Recent Advances in Pharmacological Intervention of Osteoarthritis: A Biological Aspect

**DOI:** 10.3389/fphar.2021.772678

**Published:** 2021-11-23

**Authors:** Jinxia Deng, Zhixian Zong, Zhanpeng Su, Haicong Chen, Jianping Huang, Yanru Niu, Huan Zhong, Bo Wei

**Affiliations:** ^1^ Affiliated Hospital of Guangdong Medical University, Guangdong Medical University, Zhanjiang, China; ^2^ College of Dentistry, Yonsei University, Seoul, South Korea; ^3^ Department of Stomatology, Guangdong Medical University, Zhanjiang, China

**Keywords:** osteoarthritis, DNA, RNA, exosomes, platelet-rich plasma, protein, gene

## Abstract

Osteoarthritis (OA) is a degenerative joint disease in the musculoskeletal system with a relatively high incidence and disability rate in the elderly. It is characterized by the degradation of articular cartilage, inflammation of the synovial membrane, and abnormal structure in the periarticular and subchondral bones. Although progress has been made in uncovering the molecular mechanism, the etiology of OA is still complicated and unclear. Nevertheless, there is no treatment method that can effectively prevent or reverse the deterioration of cartilage and bone structure. In recent years, in the field of pharmacology, research focus has shifted to disease prevention and early treatment rather than disease modification in OA. Biologic agents become more and more attractive as their direct or indirect intervention effects on the initiation or development of OA. In this review, we will discuss a wide spectrum of biologic agents ranging from DNA, noncoding RNA, exosome, platelet-rich plasma (PRP), to protein. We searched for key words such as OA, DNA, gene, RNA, exosome, PRP, protein, and so on. From the pharmacological aspect, stem cell therapy is a very special technique, which is not included in this review. The literatures ranging from January 2016 to August 2021 were included and summarized. In this review, we aim to help readers have a complete and precise understanding of the current pharmacological research progress in the intervention of OA from the biological aspect and provide an indication for the future translational studies.

## Introduction

**FIGURE 1:**
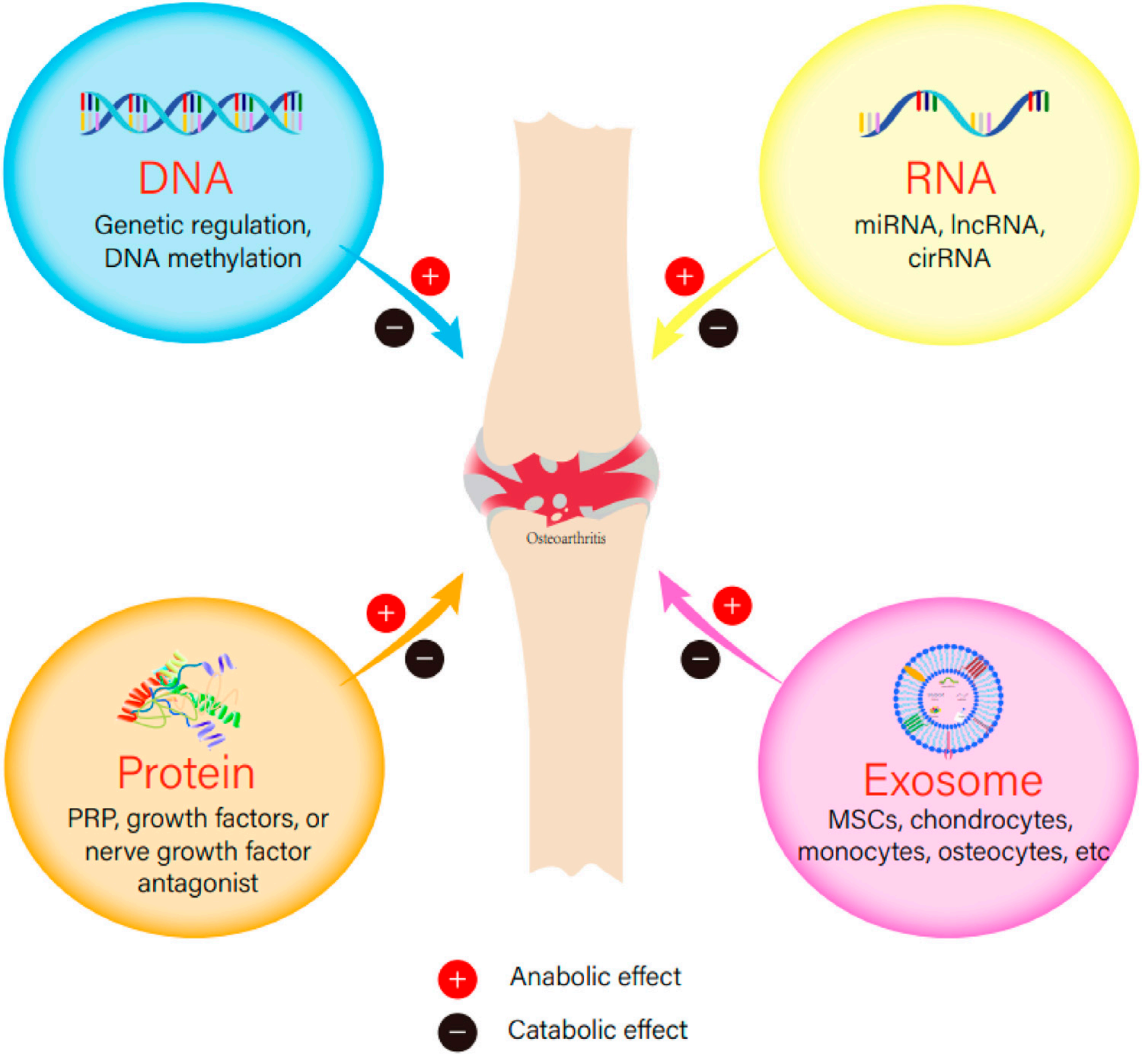
The anabolic or catabolic effect of DNA, RNA, protein, or exosome in the initiation or development of osteoarthritis.

Osteoarthritis (OA) is a degenerative chronic joint disease mainly affects the elderly, causing pain and loss of movement function. The trends of an aging population worldwide and increasing obesity are likely to make OA a leading cause of disability in the elderly (Hunter et al., 2020). Although many risk factors such as abnormal joint biomechanics, bone-mass index, joint injury, and genetic variations have been identified in the causation of OA, the etiology of OA is still poorly understood. In a traditional point of view, cartilage degradation was purely caused by mechanical imbalance (Francisco et al., 2018)_._ Currently, increasing evidence shows that OA is a complex condition, in which the whole joints, including cartilage, subchondral bone, and synovium probably, are all involved in the pathogenesis (Goldring and Goldring, 2016), among which degradation of cartilage caused by matrix proteases plays a pivotal role (Pérez-García et al., 2019). In general, OA is a disease resulting from an imbalance between catabolic and anabolic events. In recent years, biologic agents become more and more attractive as they either target specific catabolic events, such as inflammation or matrix degradation, or promote anabolic events, such as anti-inflammation or chondrogenesis. In this review, we provide an update of the current treatment strategies and recent research progress in the pharmacological intervention of OA from the biology aspect (Figure 1).

## Methods

We searched PubMed for combination of the following indexed subject headings [MeSH]: osteoarthritis, DNA, noncoding RNAs, exosomes, platelet-rich plasma, and proteins.

### Current Treatment Strategies

Clinical management for OA patients depends on their development stages of the disease. As the pathogenesis of OA is complicated, there is still no specific intervention for the treatment of OA. The primary goal for OA management is to alleviate pain and stiffness and maintain the joint function (Hermann et al., 2018). The treatment strategies for OA can be divided into three categories: nonpharmacological interventions, pharmacological interventions, and surgical interventions. Current consensus guidelines recommend the use of combination of nonpharmacological interventions, pharmacological interventions, and surgical intervention where necessary. The majority of individuals with OA can be managed successfully with a combination of nonpharmacological interventions and pharmacological interventions. However, surgical approaches should be considered at the late stages to repair the cartilage lesions or even replace the joint to regain the function.

Lifestyle modification and physical therapy are the two main nonpharmacological interventions. Body weight control in obese patients improves the symptoms and reduces the risk of symptomatic OA will develop. Exercise strengthens the muscle around the joints and maintain the stability. Physical therapy, such as pulsed electromagnetic fields (Yang et al., 2018a), extracorporeal shock wave therapy (ESWT) (Yu et al., 2017), acupuncture (Tu et al., 2021), and so on, improves the mobility and relieves the symptoms. Chondroitin sulfate and glucosamine have been used as dietary supplements.

Nonpharmacological interventions could be insufficient for many patients who develop symptomatic OA. Pharmaceutical agents, especially acetaminophen and nonsteroidal anti-inflammatory drugs, play a key role in symptom control. Other agents such as duloxetine (Weng et al., 2020), opioids, intra-articular steroid (Wijn et al., 2020), and viscosupplement injections are also approved for OA management. These drugs may effectively relieve the pain. However, many safety concerns have been raised regarding their side effects.

Surgical interventions are inevitable for many patients. Joint reservation surgeries, such as high tibial osteotomy and joint distraction, have shown symptomatic improvement (van der Woude et al., 2017). However, evidence for the long-term effectiveness is still to be confirmed. Unicompartmental knee arthroplasty (Murray and Parkinson, 2018), total knee arthroplasty (TKA) (Gademan et al., 2016), and total hip arthroplasty are widely accepted by the patients with end-stage OA.

### Recent Progress in Biological Interventions

#### DNA- or Gene-Based Therapy

DNA (Minchin and Lodge, 2019) is a double-stranded and long-chain polymer composed of four deoxynucleotides. DNA fragments with genetic information are called genes. At present, many genes are reported to be related to the occurrence and development of OA by increasing susceptibility, enhancing cartilaginous matrix degradation, preventing cartilage from repair, increasing the expression of inflammatory factors, or promoting fibroblast transformation. First, the susceptibility genes of OA mainly include ASPN (Wang et al., 2018), ADIPOQ (Shang et al., 2019), AKNA (Zhao et al., 2020a), DPEP1 (Zhang et al., 2021a), rs1065080 (Lu et al., 2019a), TLR7 (Wang et al., 2020a), RTP4 (Wang et al., 2020a), CRIP1 (Wang et al., 2020a), ZNF688 (Wang et al., 2020a), TOP1 (Wang et al., 2020a), EIF1AY (Wang et al., 2020a), RAB2A (Wang et al., 2020a), ZNF281 (Wang et al., 2020a), UIMC1 (Wang et al., 2020a), and PRKACB (Zhao, 2021). Second, the genes that promote the degradation of cartilage mainly include ADAMTS5 (Jiang et al., 2021), ADAM12 (Lv et al., 2017), JUN (Rhee et al., 2017), PTGS2 (Zhou et al., 2019a), MMP1 (Zhou et al., 2019a), MMP3 (Zhou et al., 2019a), MMP13 (Zhou et al., 2019a), AGT (Wang et al., 2020b), and rs2830585 (Zhou et al., 2019b). Third, several genes such as BMP3 (He et al., 2018), rs1799750 (Geng et al., 2018), and CHI3L1 (Song et al., 2021) show an inhibitory effect on cartilage repair. Fourth, the genes that regulate the expression of inflammatory cytokines in chondrocytes mainly include renin (Wu et al., 2019a), ACE (Wu et al., 2019a), Ang II (Wu et al., 2019a), AT1R (Wu et al., 2019a), AT2R (Wu et al., 2019a), ATF3 (Iezaki et al., 2016), PTGS2 (Lin et al., 2018; Wang et al., 2020a), CCL20 (Lin et al., 2018), CHI3L1 (Lin et al., 2018), LIF (Lin et al., 2018), CXCL8 (Lin et al., 2018), and CXCL12 (Lin et al., 2018). Last but not least, COL6A3/ACTG1 (Li et al., 2020a) and fibronectin1 (FN1) (Wu et al., 2020a) were found involved in fibroblast transformation. Although many catabolic genes have been found, there are very limited key anabolic genes that can promote the proliferation or differentiation of chondrocytes or encode key anchoring collagen molecules and the corresponding genes including GDF5 (Sun et al., 2021), Gas7 (Zhong et al., 2020), PRELP (Li et al., 2019a), TGF-β (Tao et al., 2016), SOX9 (Tao et al., 2016), and COL9A1 (Durand et al., 2020).

Genetic modification of joints has been achieved in preclinical models by *ex vivo* and *in vivo* strategies using a variety of vectors (Evans et al., 2018). Delivering genes from the body to the joints through direct intra-articular injection is a feasible way to speed up treatment. However, many vectors are inflammatory, immunogenic, or unsafe or provide only short-term transgene expression after successfully transferring cells into joint tissues. In order to solve this problem, an ideal delivery vector *in vivo* has been discovered; it is the adeno-associated virus (AAV), which is safer, more effective, and less immunogenic than other vectors (Evans et al., 2018). In addition, AAV also prolongs the expression time of transgenes in joints. When injected into the joint, the recombinant AAV will transduce synovial lining cells and chondrocytes at the thickness of the articular cartilage (Watson Levings et al., 2018). Besides the genetic regulation, epigenetic regulations, such as DNA methylation, may be also involved in OA pathology. Hypermethylation leads to a decrease in the expression of COL9A1, destroys the integrity of cartilage, and promotes the development of OA (Miranda-Duarte, 2018). SOX9 is a key transcription factor for cartilage formation in chondrocytes. The DNA methylation of SOX9 gene promoter in chondrocytes of patients with OA increases. This increase in methylation reduces the binding affinity of transcription factors, thereby reducing the expression of SOX9 in OA chondrocytes (He et al., 2020a). The DNA methyltransferases could be the potential targets to the treatment of OA in the future.

#### Noncoding RNA-Based Therapy

As mentioned, many studies in OA have focused on the epigenetic regulation of its pathogenesis and potential targets for therapy, specifically noncoding RNA (ncRNA). Human genome is estimated to contain ∼2% protein-coding RNA, whereas a vast majority of the genome comprises ncRNA. These ncRNAs, such as microRNA (miRNA), long noncoding RNA (lncRNA), and circular RNA (circRNA), are involved in the pathological development of OA, which can be used as diagnostic and therapeutic markers for OA progression and prognosis. Recent preclinical evidence shows that many ncRNAs can directly affect the expression of key genes involved in OA, which have great translational potential in OA treatment (Duan et al., 2020). Future research on elucidating the role of ncRNAs will also help in better understanding the etiology of OA. In particular, research and development of therapeutic targets for OA provide important clues (Cong et al., 2017). However, studies also report that many ncRNAs are considered the critical elements in cancer development (He et al., 2021a). Sufficient preclinical safety inspections should be performed before clinical use (Xie et al., 2020a).

##### MiRNA

Among those ncRNAs, miRNAs are most popular in recent years, with approximately 22 nucleotides, functioning in RNA silencing and posttranscriptional regulation of gene expression. Many studies have reported that several miRNAs could play an important role in regulating bone and cartilage homeostasis (Shen et al., 2019) (Table 1), through regulating the signaling pathways involved in extracellular matrix (ECM) degradation, apoptosis or hypotrophy of chondrocytes, or synovial inflammation.

**TABLE 1:** miRNA and the targets in osteoarthritis.

Functions	Effects	MiRNAs	Targets	References
Negative regulation	Inhibit chondrocyte proliferation	miR-21	GDF-5	Sekar (2021)
	Promote osteoclast formation	miR-21	Unknown	Sekar (2021)
	Promote chondrocyte apoptosis	miR-146a	SMAD4	Malemud (2018)
		miR-1236	PIK3R3	Wang et al. (2020c)
		miR-34a	Visfatin (NF-ΚB)/ADAMTS-4	Cheleschi et al. (2019)
		miR-181a	GPD1L	Zhai et al. (2017)
		MiR-155	GPD1L	Zhai et al. (2017)
		miR-384-5p	SOX9	Zhang et al. (2018a)
		miR-9	Sirtuin-1	D'Adamo et al. (2017)
		miR-335-5P	HBP1	Lu et al. (2021)
		miR-107	TRAF3	Zhao et al. (2019)
	Promote inflammation	miR-149-5p	AGT	Wang et al. (2020b)
	Increase matrix degradation	miR-33a	TGF-β1/Akt/SREBP-2	Ghafouri-Fard et al. (2021)
		miR-483-5p	HDAC4, Matn3/Timp2	Wang et al. (2019a)
		miR-101	SOX9	Chu et al. (2019)
	Promote cartilage degradation	miR-141/200c	SIRT1	Ji et al. (2020a)
		miRNA 218-5p	PIK3C2A	Lu et al. (2017)
		miR-146b	Alpha-2-macroglobulin (A_2_M)/SOX5	Liu et al. (2019b)
		miR-21-5p	FGF18	Wang et al. (2019b)
		miR-98	Bcl-2	Wang et al. (2017b)
	Inhibit chondrocyte differentiation	miR-582-5p	Runx2	Wang et al. (2019c)
		miR-324-5p	GLI1 and SMO	Woods et al. (2019)
Positive regulation	Promote chondrocyte proliferation	miR-132	PTEN/PI3K/AKT	Zhang et al. (2021c)
		miR29a	MMP-13/ADAMTS-5	Komori (2016)
		miR-138	NEK2	Xu et al. (2019)
		miR-4784	Col2a1/MMP-3	Liu et al. (2018)
		miR-210	HIF-3α	Zhao et al. (2020c)
		miR-101	Sox9/Runx2	Gao et al. (2019)
		miR-210-3p	SOX9/COLII	Yang et al. (2021b)
	Promote cartilage regeneration	miR-149-5p	FUT-1	Çelik et al. (2019)
	Inhibit chondrocyte apoptosis	miR-766-3P	AIFM1	Li et al. (2020b)
		miR-132	PTEN/PI3K/AKT	Zhang et al. (2021c)
		miR-582-3p	YAP1	He et al. (2020b)
		miR-455-3p	PI3K/AKT	Wen et al. (2020)
		miR-138	NEK2	Xu et al. (2019)
		miR-93-5p	TCF4	Xue et al. (2019)
	Repression of chondrocyte autophagy	miR-130a	HOTAIR lncRNA	Hu et al. (2019)
	Inhibit osteoclast formation	miR-125b	Unknown	Yoshiko and Minamizaki (2020)
	Inhibit the degradation of cartilage	miR-221	SDF1/CXCR4	Zheng et al. (2017)
	Decrease metabolic enzyme activity	miR-1	FZD7	Xie et al. (2020a)
	Suppress inflammation	miR-582-3p	YAP1	He et al. (2020b)
		miR-335-5p	3-MA	Zhong et al. (2019)
		miR-106a5p	GLIS3	Ji et al. (2018)
	Inhibit ECM degradation	miR-582-3p	YAP1	He et al. (2020b)
		miRNA-140	MMP-13/ADAMTS-5	Si et al. (2017)
		miR-145	MKK4	Hu et al. (2017)
	Enhance cartilage repair	mi-107	HMGB-1	Lin et al. (2019)
	Inhibit the destruction of articular cartilage	miR-204	Runx2	Huang et al. (2019a)

##### LncRNA

lncRNAs are another type of ncRNAs that are longer than 200 nucleotides (Zhang et al., 2021b). LncRNA–RNA interaction controls mRNA translation and degradation, or as silent miRNA sponges. They are also regarded as important regulators of cartilage development (Table 2). The anti-OA mechanism of lncRNA may be achieved by competitively binding miRNA, reducing the binding of miRNA and downstream genes, and increasing the transcription and expression of downstream genes (Wu et al., 2019b).

**TABLE 2:** lncRNAs and the targets in osteoarthritis.

Functions	RNAs	Target	References
Negative regulation	MIAI	miR-132	Li et al. (2019b)
	DANCR	miR-216a-5p/JAK2	Zhang et al. (2018b)
	TM1P3	miR-22	Li et al. (2019c)
	CTD-2574D22.4	Unknown	Li et al. (2019d)
	CAIF	miR-1246	Qi et al. (2019)
	TNDSF10	miR-376-3p/FGFR1	Huang et al. (2019b)
	LOC101928134	IFNA1	Yang et al. (2019a)
	CASA2	Unknown	Huang et al. (2019c)
	CHRF	microRNA-146a	Yu et al. (2019)
	Nespas	miR-291a-3p	Park et al. (2019)
	H19	miR-130a	Hu et al. (2019)
	THRIL	microRNA-125b	Liu et al. (2019c)
	TUG	miR-195	Tang et al. (2018)
	P21	miR-130b	Han and Liu (2018)
	CIR	miR-27	Li et al. (2017a)
	PVT1	miR-488-3p	Li et al. (2017b)
	XIST	miR-211	Li et al. (2018a)
	MBNL1-AS1	KCNMA1	Li et al. (2018b)
	HOTAIR	miR-17-5p	Hu et al. (2018)
		miR-130a-3p	He and Jiang (2020)
	FAS-AS1	MMP1/MMP13/COL2A1	Zhu et al. (2018)
	TMSB4	miRNA-152	Liu et al. (2016)
	HOTTIP	Unknown	He et al. (2021b)
	LINC02288	miR-374a-3p	Fu et al. (2021)
	LINC01534	miR140-5p	Wei et al. (2019)
	MSR	miR-152	Liu et al. (2016)
	PART1	miR-373-3p/SOX4	Zhu and Jiang (2019)
	GAS5	miR-34a/Bcl-2	Ji et al. (2020b)
	NEAT1	miR-193a-3p/SOX5	Liu et al. (2020)
Positive regulation	FOXD2-AS1	miR-27a-3p	Wang et al. (2019d)
		miR-206	Cao et al. (2018)
	ANCR	TGF-β1	Li et al. (2019e)
	ANRIL	miR-122-5p/DUSP4	Li et al. (2019f)
	DILC	IL-6	Huang et al. (2019d)
	DNM3OS	miR-126/IGF1	Ai and Yu (2019)
	MIR4435-2HG	Unknown	Xiao et al. (2019a)
	SNHG1	MAPK/NF-κB	Lei et al. (2019)
	HULC	miR-101	Chu et al. (2019)
	HOTAIRM1-1	miR-125b/BMPR2	Xiao et al. (2019b)
	PACER	Unknown	Jiang et al. (2019)
	PART1	miR-590-3p/TGFBR2/SMAD3	Lu et al. (2019b)
	MEG3	miR-93	Chen et al. (2019)
		miR-16	Xu and Xu (2017)
	LINC00341	miR-141	Yang et al. (2019b)
	ATB	miR-223	Ying et al. (2019)
	PMS2L2	miR-203	Li et al. (2019g)
	MALAT1	miR-150-5p	Zhang et al. (2019)
	ROR	HIF1α/p53	Yang et al. (2018b)
	ZFAS1	Wnt3a	Ye et al. (2018)
	GACAT3	IL-6/STAT3	Li et al. (2018c)
	UFC1	miR-34a	Zhang et al. (2016)
	NKILA	miR-145/SP1/NFκB	Xue et al. (2020)

##### CircRNA

CircRNA is a covalently closed circRNA molecule that contains exon sequences and is spliced at the canonical splicing site (Tam et al., 2019), functioning as miRNA sponges or competing endogenous RNAs that naturally sequester and competitively inhibit miRNA activity. CircRNAs also emerge as a new player in the development of OA through mechanisms such as interfering chondrocyte proliferation and apoptosis, regulating ECM degradation, and inflammation (Yang et al., 2020) (Table 3).

**TABLE 3:** CircRNAs and the targets in osteoarthritis.

Functions	RNAs	Target	References
Negative regulation	CircRNA-UBE2G1	miR-373/HIF-1a	Chen et al. (2020a)
	Circ_0136474	miR-127-5p/MMP-13	Li et al. (2019h)
	CircPSM3	miRNA-296-5p	Ni et al. (2020b)
	has_Circ_0005105	miR-26a/NAMPT	Wu et al. (2017b)
	hsa_Circ_0032131	unknown	Wang et al. (2019e)
	hsa_Circ_0104873	Unknown	Yu et al. (2018)
	hsa_Circ_0104595		
	hsa_Circ_0101251		
	CircRNA-CDR1as	miR-641/FGF-2	Zhang et al. (2020)
	CircRNA_Atp9b	miR-138-5p	Zhou et al. (2018)
	CircRNA.33186	miR-127-5p/MMP-13	Zhou et al. (2019c)
	CircGCN1L1	miR-330-3p/TNF-α	Zhu et al. (2020)
	Circ-SERPINE2	miR-1271/ERG	Tam et al. (2019)
	CiRS-7	miR-7	Zhou et al. (2019d)
	CircHYBID	miR-29b-3p/TGF-β1	Liao et al. (2021)
	Circ-SPG11	miR-337-3p/ADAMTS5	Liu et al. (2021)
	Circ-CSNK1G1	miR-4428/FUT2	Xiao et al. (2021)
Positive regulation	CircVCAN	NF-κB	Ma et al. (2020)
	Circ9119	miR-26a/PTEN,	Chen et al. (2020b)
	hsa_Circ_0045714	miR-193b/IGF-1R	Li et al. (2017c)
	hsa_Circ_0020014	Unknown	Wang et al. (2020d)
	CircPDE4D	miR-103a-3p/FGF18	Wu et al. (2021)

#### Protein-Based Therapy

The protein currently used in clinical practice is mainly platelet-rich plasma (PRP) (Szwedowski et al., 2021). PRP is an autologous plasma preparation rich in platelets whose plasma concentration is higher than the normal concentration in whole blood. The basic principle of therapeutic potential of high-concentration platelets is based on their ability to provide superphysiological amounts of essential growth factors to provide regenerative stimulation that can promote tissue repair. PRP preparations need to be activated before use (Gentile et al., 2020). Intra-articular injections of PRP may be an effective alternative treatment to pain killers for knee OA (Rajan et al., 2020). It significantly promoted the proliferation of chondrocytes, decreased apoptosis, and increased autophagy by regulating the markers including FOXO1, FOXO3, and HIF-1 in osteoarthritic chondrocytes (Moussa et al., 2017). The concentration of white blood cells during the leukocyte-rich PRP (LR-PRP) preparation will affect its efficacy (Yaşar Şirin et al., 2017). It is reported that compared with the LR-PRP, the leukocyte-poor PRP (LP-PRP) has an effect on improving the proliferation of chondrocytes. The lubricating property of hyaluronic acid (HA) facilitates the movement of joints. And a combination of HA and PRP (HA–PRP) (Zhao et al., 2020b) could exert a beneficial synergistic effect for OA treatment. However, up until now, the preparation method and the components of PRP have still not been standardized, making the efficacy of PRP therapy to be inconclusive.

In addition to PRP, the proteins currently studied include nerve growth factor antibody (Grässel and Muschter, 2020) or its antagonists (Denk et al., 2017), fibroblast growth factor (FGF) (Xie et al., 2020b), insulin-like growth factor–binding proteins (IGFBP) (Tanaka et al., 2021), growth and differentiation factor 5 (Kania et al., 2020), Wnt16 (Tong et al., 2019), low-density lipoprotein receptor–related protein 5 (Wu et al., 2017a), neuropeptide Y (NPY) (Kang et al., 2020), and so on. Among the proteins, fasinumab (Dakin et al., 2020), tanezumab (Berenbaum et al., 2020), sprifermin (Eckstein et al., 2020), teriparatide (Apostu et al., 2019), and so on, have shown various effects on the management of OA in clinical trials. Nerve factor antibodies and their antagonists, fasinumab and tanezumab, can improve pain, and the antagonists have the most significant effect. Tanezumab can easily lead to rapidly progressive OA. FGF, GDF5, Wnt16, NPY, sprifermin, and teriparatide are related to cartilage repair. IGFBP is related to cartilage matrix synthesis. The binding of low-density lipoprotein receptor–related protein and sclerostin can inhibit the degradation of normal chondrocytes, but it does not seem to have such an effect in OA. The specific reason is not clear.

Recently, histone modifications have been recognized as another important epigenetic regulation in OA-related genes. LSD1 KDM4B, KDM6A, KDM6B, EZH2, and DOT1L were reported to be the major epigenetic regulators in OA onset and progression through their methyltransferases and demethylase activities by binding to the OA-related gene (e.g., Runx2, Nfat1, and Sox9) promoters or by interplaying with OA-associated signaling transduction pathways (Sacks et al., 2018). Modified histone domains have thus become epigenetic signatures, which will either mark for gene activation or gene repression. The role of methyltransferases and demethylase in epigenetic regulations also indicate they could be potential targets for the management of OA.

#### Exosomes

Exosomes are small, single-membrane, secreted organelles with a diameter of approximately 30–200 nm. They have the same topological structure as cells and are rich in selected proteins, lipids, nucleic acids, and glycoconjugates (Pegtel and Gould, 2019). Exosomes mainly mediate cell–cell communication through direct membrane fusion or protein–protein interaction (Wu et al., 2020b). The source of exosomes comes in many forms (Ni et al., 2020a), including peripheral blood (Chang et al., 2018), synovial fluid (Gao et al., 2020), mesenchymal stem cells (Tofiño-Vian et al., 2018), embryonic stem cells (Wang et al., 2017a), vascular endothelial cells (Yang et al., 2021a), dental pulp stem cells (Lin et al., 2021), monocytes (Bai et al., 2020), amniotic fluid stem cells (Beretti et al., 2018), chondrogenic progenitor cells (Toh et al., 2017), chondrocytes (Zheng et al., 2019), PRP (Liu et al., 2019a), osteocytes (Lyu et al., 2020) (Table 4), and so on. Exosomes with different origins may have different functions. Exosomes in the joint microenvironment are involved in the development of OA. Most therapeutic exosomes may have an anabolic effect by promoting expression of chondrogenic markers or cartilage ECM or exert an effect by inhibiting inflammation, hypertrophy, or apoptosis of chondrocytes (Zhou et al., 2020) showing great potential for OA therapy.

**TABLE 4:** Exosomes in the treatment of OA.

Functions	Origins	Mechanisms
Catabolic effect	Synovial fluid (Gao et al., 2020), vascular endothelial cells (Yang et al., 2021a)	Recruit inflammatory cells (Gao et al., 2020), inhibit cartilage proliferation (Gao et al., 2020), promote joint degeneration (Gao et al., 2020), or induce chondrocyte apoptosis (Yang et al., 2021a)
Anabolic effect	Mesenchymal stem cells (Tofiño-Vian et al., 2018), embryonic stem cells (Wang et al., 2017a), dental pulp stem cells (Lin et al., 2021), monocyte (Bai et al., 2020), amniotic fluid stem cells (Beretti et al., 2018), chondrogenic progenitor cells (Toh et al., 2017), chondrocytes (Zheng et al., 2019), platelet-rich plasma (Liu et al., 2019a), osteocytes (Lyu et al., 2020)	Reduce production of catabolic enzymes (Tofiño-Vian et al., 2018), promote chondrocytes to express cartilage ECM (Wang et al., 2017a; Kim et al., 2020; Guillén et al., 2021), promote chondrocyte differentiation (Bai et al., 2020), promote proliferation of chondrocytes (Liu et al., 2019a; Lyu et al., 2020), inhibit chondrocyte apoptosis (Liu et al., 2019a; Lyu et al., 2020; Lin et al., 2021), regulate immune response (Zheng et al., 2019), or inhibit expression of inflammatory cytokines (Toh et al., 2017; Beretti et al., 2018; Tofiño-Vian et al., 2018; Kim et al., 2020; Qiu et al., 2021)

Sustained-release drug delivery systems have been developed by a combination of exosomes and tissue engineering strategies, showing great promising results in recent research by delivering targeted drug or nucleic acids for regenerative medicine (Akbari et al., 2020). However, because of complexity in the components and rare understanding of their functions, exosomes remain challenges for clinical applications (Zhou et al., 2020).

## Literature Analysis

**FIGURE 2:**
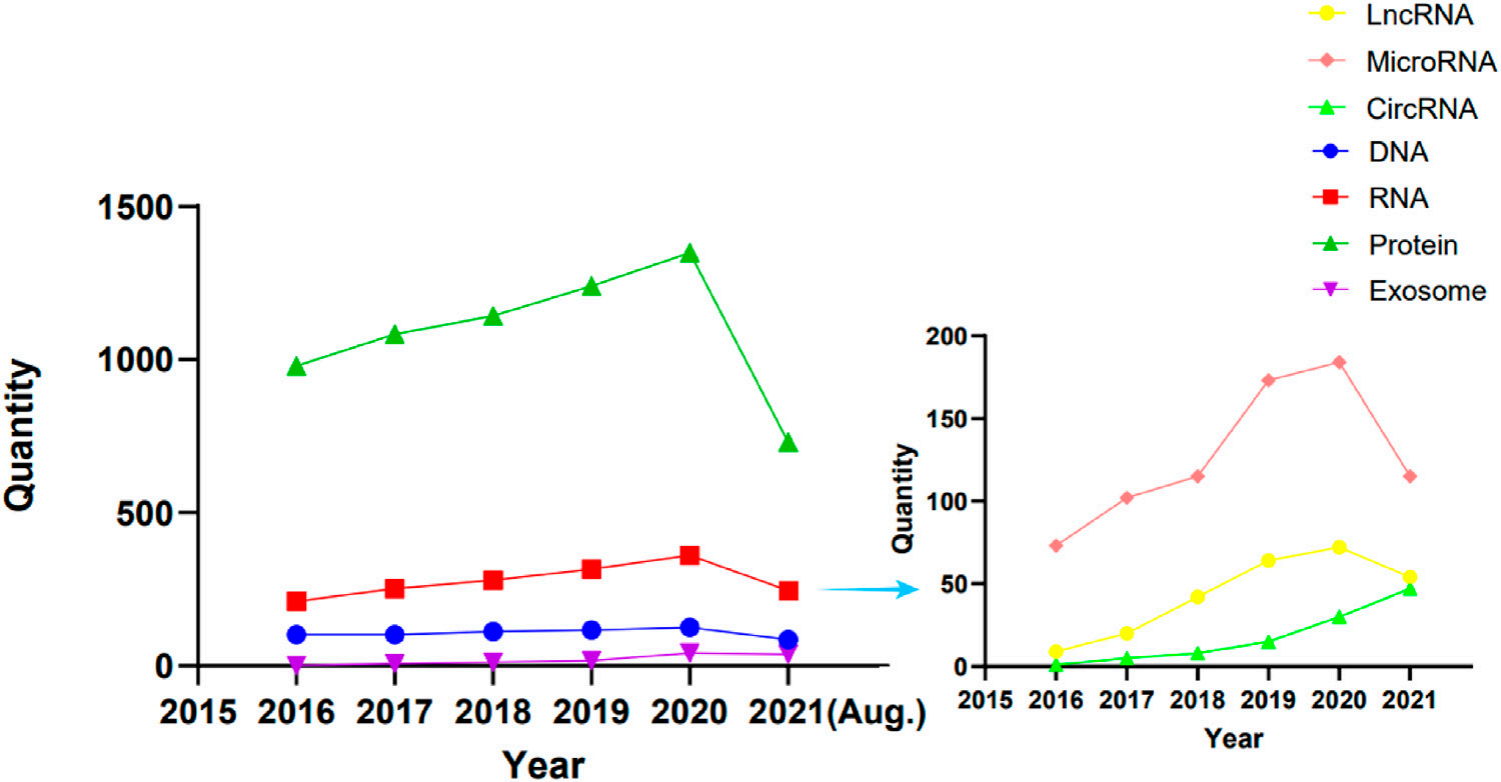
Publication trends in biologics from January 2015 to August 2021.

In order to analyze the research trends in the field of OA treatment using the biologic agent in recent years, we have reviewed relevant literature on DNA, RNA, protein, and exosome in the past 5 years on PubMed and also subdivided RNA into circRNA, lncRNA, and miRNA. We present a graphic (Figure 2) and the corresponding table (Supplementary Table S1) to show the literature trend in the past 5 years from January 2016 to August 2021. From the results, we can see that the number of articles of each type of biological agent has increased throughout the past 5 years. Among the four types of biologic agents, the most abundant research on proteins was found, followed by RNA, then DNA, and finally exosomes. Within RNA, miRNA has been studied most intensively, followed by lncRNA, and finally circRNA. This result shows that the research on proteins and RNA is relatively mature, but DNA and exosomes are new highlights in recent years. Within RNA, there are relatively many studies on miRNA and relatively fewer studies on lncRNA and circRNA. Therefore, DNA, exosomes, lncRNA, and circRNA may all become new research hotspots.

## Discussion

DNA, RNA, and protein described in this article have shown various regulatory effects on the pathological process of OA. Some of those are expected to become targets in terms of diagnosis and treatment of OA. In general, the effects of biologic agents are divided into two aspects: catabolic or anabolic effect by deteriorating or preventing OA occurrence or development. The catabolic effect is mainly to recruit inflammatory cells, inhibit chondrocyte proliferation, accelerate matrix degradation, or induce cell apoptosis. In opposite, the anabolic effect is mainly to reduce the production of catabolic enzymes, promote the proliferation of chondrocytes, inhibit chondrocyte apoptosis, promote the expression of ECM, or inhibit the expression of inflammatory factors. The main pathways involved in OA treatment are NF-κB, Notch, Wnt/β-catenin, TGF-β, Erk, p38 MAPK, JAK2/STAT3, and so on. At present, most researches on biologic agents are *in vitro* experiments or animal model experiments. There are still many obstacles to overcome for the biologics agents: (1) safety concern is the first to be considered when applying viral vectors to deliver plasmids, ncRNAs, which may bind to multiple targets; and exosomes and proteins, which may result in immunoresponse and disease transmission; (2) efficacy of most of the biologic agents in OA therapy is various and still yet to be verified; (3) heterogeneity of disease may also affect the therapeutic outcomes. With the advancement of molecular biotechnology in future research, translation research should be considered to address the limitations before clinical trials.

## Conclusion

We have reviewed a wide spectrum of biologic agents in OA therapy, including DNA, RNA, protein, and exosomes, which provide an insight in finding potential therapeutic targets. Although significant progress has been made in this field, translational research is needed to further address the safety concerns, various efficacies, and heterogenetic of OA.

## Abbreviations

CircRNA, circular RNA; FGF, fibroblast growth factor; HA–PRP, hyaluronic acid–platelet-rich plasma; IGFBP, insulin-like growth factor–binding proteins; lncRNA, long noncoding RNA; LP-PRP, leukocyte-poor–platelet-rich plasma; LR-PRP, leukocyte-rich–platelet-rich plasma; miRNA, microRNA; OA, osteoarthritis; PRP, platelet-rich plasma; TGF-β, transforming growth factor β.
