# Supplementary material for: Recent Advances in Pharmacological Intervention of Osteoarthritis: A Biological Aspect
Source: Front Pharmacol. 2021 Nov 23;12:772678. doi: 10.3389/fphar.2021.772678 (PMC8649959; doi:10.3389/fphar.2021.772678)
Supplement: Supplementary file 1 [file DataSheet1.PDF]

Table S1. Changes in biologics in recent years

Year / Quantity / Type	2016	2017	2018	2019	2020	2021(Aug.)
DNA	102	102	112	117	125	86
RNA	210	252	279	315	360	246
Protein	979	1083	1143	1241	1349	731
Exosome	1	7	12	17	42	38
LncRNA	9	20	42	64	72	54
MicroRNA	73	102	115	173	184	115
CircRNA	1	5	8	15	30	47
